# Facemap: a framework for modeling neural activity based on orofacial tracking

**DOI:** 10.1038/s41593-023-01490-6

**Published:** 2023-11-20

**Authors:** Atika Syeda, Lin Zhong, Renee Tung, Will Long, Marius Pachitariu, Carsen Stringer

**Affiliations:** HHMI Janelia Research Campus, Ashburn, VA USA

**Keywords:** Neural encoding, Neural circuits, Machine learning

## Abstract

Recent studies in mice have shown that orofacial behaviors drive a large fraction of neural activity across the brain. To understand the nature and function of these signals, we need better computational models to characterize the behaviors and relate them to neural activity. Here we developed Facemap, a framework consisting of a keypoint tracker and a deep neural network encoder for predicting neural activity. Our algorithm for tracking mouse orofacial behaviors was more accurate than existing pose estimation tools, while the processing speed was several times faster, making it a powerful tool for real-time experimental interventions. The Facemap tracker was easy to adapt to data from new labs, requiring as few as 10 annotated frames for near-optimal performance. We used the keypoints as inputs to a deep neural network which predicts the activity of ~50,000 simultaneously-recorded neurons and, in visual cortex, we doubled the amount of explained variance compared to previous methods. Using this model, we found that the neuronal activity clusters that were well predicted from behavior were more spatially spread out across cortex. We also found that the deep behavioral features from the model had stereotypical, sequential dynamics that were not reversible in time. In summary, Facemap provides a stepping stone toward understanding the function of the brain-wide neural signals and their relation to behavior.

## Abstract (web summary)

Facemap is a data analysis framework for tracking keypoints on mouse faces and relating them to large-scale neural activity. Both of these steps use state-of-the-art convolutional neural networks to achieve high precision and fast processing speeds.

## Main

Neurons across the brain are constantly active, even in the absence of external sensory stimuli or a behavioral task^1,2^. This ongoing, spontaneous neural activity is driven by the spontaneous behaviors of the animal, such as running, head movements and whisking in mice^3–9^, tail movements in zebrafish^10^ and body movements in flies^11–13^. In mice, different neurons were best explained by different combinations of orofacial behaviors, such as whisking, sniffing and grooming, showing that multidimensional representations of behavior exist across the brain^14–17^. These multidimensional behavioral representations persist during presentations of sensory stimuli^14^ and decision-making tasks^18–20^.

Despite the widespread presence of behavioral signals across the brain, their role and function remains poorly understood. To make progress in understanding these neural signals, it is important to develop better computational models. This requires progress in the following two areas: (1) better quantification of orofacial behavior and (2) better models of the influence of behavior on neural activity.

To quantify behavior, previous studies took advantage of the stability of the head-fixed experimental setup to compute low-dimensional features of the raw behavior movies, either using principal components (PCs) of the movies^14,17,20^, or using autoencoders fit to the movies^21,22^. Although movie PCs are easy to compute, the resulting features are hard to interpret. Another common approach for quantifying orofacial movements is whisker tracking, which can provide specific and interpretable information about whisker motion^23–26^. However, previous approaches for whisker tracking required trimming the other whiskers and/or whisker painting, which may alter mouse behavior, and they also required a high-speed overhead camera, which may be unavailable in many experimental setups. An alternative approach is markerless pose estimation or keypoint tracking. Several algorithms exist for general keypoint tracking in animals^27–31^, but none of these tools have specialized methods for tracking orofacial movements.

Similarly, better models are needed to account for the influence of behavior on neural activity. Previous studies used simple approaches like reduced-rank regression (RRR) or ridge regression^14,20^. These models are linear and do not take into account temporal dynamics. Therefore, they are unlikely to capture the full influence of time-varying, multidimensional behavior on neural activity.

To address these shortcomings, we developed two new algorithms as follows: a keypoint tracker and a single neuron prediction model, both of which we make available in Facemap. Both algorithms are powered by deep neural networks. To track orofacial behaviors, we developed a pose estimation tool that tracks 13 distinct keypoints on the mouse face from variable camera views. Our pose estimation tool is more accurate than the best existing method (DeepLabCut), and it is also twice as fast, thus providing a viable option for online behavioral tracking. On new data, the Facemap tracker requires only ten new labeled frames for near-optimal performance. We also developed a multilayer neural network that is optimized to predict neural dynamics from orofacial behaviors. Compared to previous methods, this approach can predict almost twice as much neural variance for neurons in visual cortex. Furthermore, the model learns deep behavioral features that have highly-structured state dynamics, which we inferred using a hidden Markov model (HMM). Hence, Facemap can be used to obtain insights into both the structure and influence of orofacial behaviors on neural activity, thus providing a stepping stone toward understanding the function of the brain-wide behavioral signals.

## Results

### Fast and accurate tracker for mouse orofacial movements

**Fig. 1:**
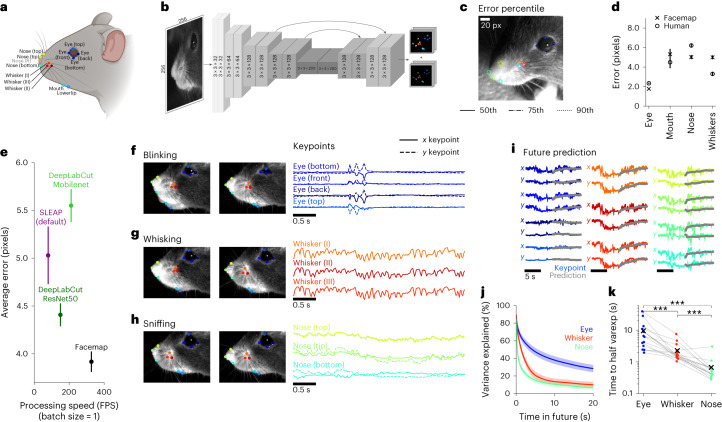
Fast and accurate mouse orofacial keypoint tracking. **a**, A total of 13 distinct keypoints selected for tracking the eye, mouth, whiskers and nose on the mouse face, illustration created with BioRender.com. **b**, Architecture of the Facemap network, a U-Net style convolutional neural network. **c**, The error percentiles across test frames from a new mouse, where error is defined as the Euclidean distance between the ground-truth label and the prediction. **d**, Summary of Facemap performance on test data for different subgroups of keypoints. Human error shown for a subset of the test frames labeled in two different sessions by a human annotator. Error bars represent s.e.m., *n* = 400, 95, 361 and 300 keypoint labels for eye, mouth, nose and whiskers, respectively, across 100 test frames. **e**, The average error, in pixels, and processing speed, in video frames processed per second, of the Facemap tracker compared with other pose estimation tools. Error bars represent s.e.m., *n* = 1,156 keypoint labels. **f**–**h**, Traces of *x* and *y* coordinates of keypoints during different orofacial behaviors. **i**, Prediction of keypoint traces into the future (test data). **j**, Variance explained of future prediction at different time lags, summarized for each face region. Error bars represent s.e.m., *n* = 16 recordings. **k**, Decay time to 50% of variance explained at 20 ms timelag. The ‘*x*’ represents the average. Two-sided Wilcoxon signed-rank test, ***P < 0.001 (eye versus whisker, *P* = 3.05 × 10^−^^5^; eye versus nose, *P* = 3.05 × 10^−^^5^; whisker versus nose, *P* = 1.53 × 10^−4^).

We start by describing a neural network model for keypoint tracking on the mouse face, the Facemap tracker. As a first step, we chose several well-defined keypoints that could track various orofacial movements (Fig. 1a). To capture whisking, we tracked three whiskers that are visible from most camera views, labeling the points at the base of the whiskers. To capture sniffing, we tracked four nose-related keypoints (bottom, top, tip and right-bottom, when in view). To capture mouth movements, when the mouth was in view, we tracked two mouth keypoints (mouth and lower lip). To capture eye movements, such as blinking, we tracked the four corners of the eye (bottom, top, front and back). We did not track the pupil, because it is completely dilated and untrackable in darkness, and also because it is easier to track with simpler methods^14^.

**Extended Data Fig. 1:**
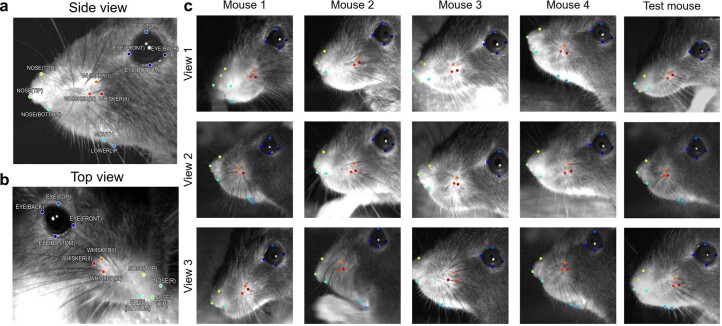
Mouse face keypoints from different camera views. Keypoints labels and sample images shown from training and test set. **a**, Side view recording of a mouse face showing eye, whiskers, nose and mouth keypoints. **b**, Top view recording of mouse face in **a** showing eye, whiskers and nose keypoints from a different view. **c**, Mouse face recordings from different camera views for training samples and test samples (last column). A total of 2,400 training frames were used from mice shown in **c** and other mice (not shown), and 100 frames from different views of a new mouse used as the test set.

Our goal was to build a model that would generalize well to new data. To achieve this goal, we collected a dataset of short mouse face videos from many different mice with the camera setup at several different angles. From this dataset of 16 mice and 53 video recordings at different views, we manually annotated 2,500 frames (Extended Data Fig. 1). We used 2,400 frames for training the network and set aside a test set consisting of 100 frames from multiple views of a new mouse.

Unlike more general approaches, like DeepLabCut and SLEAP^27,31^, we only require our tracker to perform well on specific keypoints from the mouse face. Thus, we hypothesized that a minimal ‘U-Net’-style neural network^32^ would be sufficient for the task while providing faster tracking compared to the existing, bigger models (Supplementary Table 1). Similar to DeepLabCut, which in turn is based on DeeperCut^33^, the Facemap tracker takes as input an image and outputs a set of downsampled probability heatmaps and location refinement maps to predict the *x* and *y* coordinates for each keypoint (Fig. 1b). The likelihood values of the model prediction were used to filter the traces and remove outliers (Methods^27^). The Facemap tracker was implemented from scratch using the neural network software PyTorch^34^, which is a popular and easy-to-use alternative to the TensorFlow framework^35^ used by DeepLabCut and SLEAP.

**Extended Data Fig. 2:**
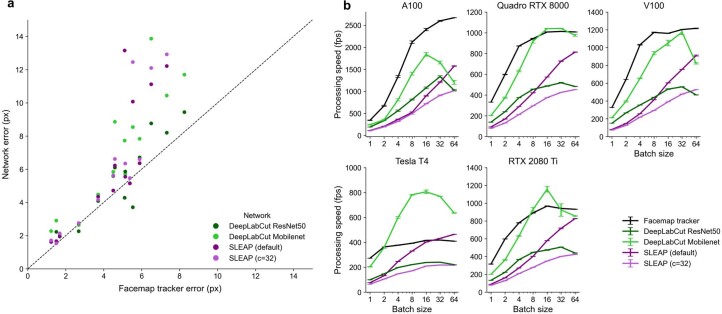
Per keypoint error and processing speed of networks using various batch sizes. **a**, Error for each keypoint, averaged across 100 test frames for each network plotted against the Facemap tracker errors. Points above the diagonal indicate keypoints for which Facemap outperformed the other networks. **b**, Processing speed of Facemap, DeepLabCut (ResNet50), DeepLabCut (Mobilenet), SLEAP (default) and SLEAP (c = 32) models for a sample image of size 256 × 256 pixels on A100 (48 slots, 40GB/slot), V100 (48 slots, 30GB/slot), RTX 2080 Ti (40 slots, 18GB/slot), Tesla T4 (48, 15GB/slot) and Quadro RTX 8000 (40 slots, 18GB/slot). Processing speed averages shown for a total of 1,024 frames across n = 10 runs, and error bars represent SEM.

The keypoint error percentiles shown on an example test frame demonstrate the accuracy of the tracking (Fig. 1c and Supplementary Video 1). To get an upper bound on the tracking performance, we manually labeled test frames twice at different orientations and compared the two sets of labels. We found that the tracker achieved human-level performance (Fig. 1d). We compared our model with current state-of-the-art tools for keypoint tracking, DeepLabCut and SLEAP^27,31,36,37^. The Facemap tracker was more accurate than the other well-performing network, DeepLabCut with the ResNet50 backbone, both in average error (3.9 versus 4.4 pixels) and for individual keypoints of the face (Fig. 1e and Extended Data Fig. 2a). Facemap also outperformed DeepLabCut with the Mobilenet backbone, SLEAP default and SLEAP’s larger network (32 channels), which had average errors of 5.6, 5.0 and 5.7 pixels, respectively (Fig. 1e and Extended Data Fig. 2a).

To compare the speed of the networks for the purpose of online tracking, we computed the processing speed using a batch size of 1 (Fig. 1e). All the networks can achieve higher speeds with larger batch sizes, but only a batch size of 1 can be used for online processing of keypoints for closed-loop experiments. The smaller size of the Facemap tracker network provided a much faster processing speed of 327 Hz on a V100 GPU compared to DeepLabCut’s ResNet50 network (150 Hz), DeepLabCut’s Mobilenet network (211 Hz), SLEAP’s default network (80 Hz) and SLEAP’s larger network (*c* = 32; 72 Hz). Across different GPU types, Facemap consistently demonstrated the fastest processing speed (Supplementary Table 2). We also benchmarked the processing speed of the Facemap tracker at larger batch sizes and found that it was as fast or faster than all other networks across GPUs except for the Tesla T4 GPU, where DeepLabCut Mobilenet was the fastest (Extended Data Fig. 2b). Therefore, Facemap is the fastest orofacial tracker with state-of-the-art performance, which enables its use in closed-loop experiments with high frame rates.

The keypoints tracked by Facemap captured recognizable orofacial behaviors, such as blinking (Fig. 1f), whisking (Fig. 1g) and sniffing (Fig. 1h), in addition to other orofacial behaviors. In the neural recordings, the camera view in Fig. 1c was used, so mouth keypoints were not included in the analyses as they were not visible. Therefore, for the rest of this study, we use the eye, whisker and nose keypoints to characterize the aspects of behavior and neural activity. To start, we investigated the timescales of the orofacial keypoints. To do this, we built an autoregressive model to predict the position of each keypoint in the future (prediction shown in Fig. 1i). The variance explained by the model on test data decayed as a function of time into the future (Fig. 1j). The predictability of the nose keypoints decayed fastest (~1 s), followed by the whiskers (~3 s) and eye keypoints (~10 s; Fig. 1k). This was surprising because whisking was the fastest behavior observed in the videos (~10 Hz). However, these fast movements were pseudo-random (Fig. 1g) and hard to predict, so they did not contribute strongly to the predictability of the whisker keypoints.

### Fine-tuning the Facemap tracker on new videos

**Fig. 2:**
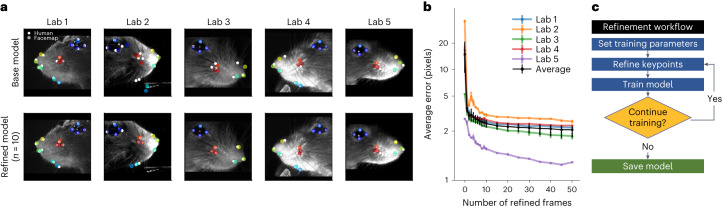
Keypoint tracking on mice from other labs by fine-tuning the Facemap tracker. **a**, Top, keypoint predictions using the Facemap tracker’s base model (white circles) and human annotations (colored circles) on mice from new experimental setups. Bottom, keypoint predictions from the fine-tuned model trained with number of refined frames = 10. **b**, Performance of the Facemap tracker measured by average error (pixels) on test frames, as a function of the number of refined frames used for fine-tuning the base model (number of refined frames = 0 is the base model), for each lab and average test error across labs (black). There were *n* = 50 independent test frames per lab averaged, and error bars represent s.e.m. Note that the test errors in this panel are slightly lower than on the original training dataset (Fig. 1e), likely because the ground-truth labels were refined from predictions of the model. **c**, A flowchart of the refinement workflow implemented in our GUI.

**Extended Data Fig. 3:**
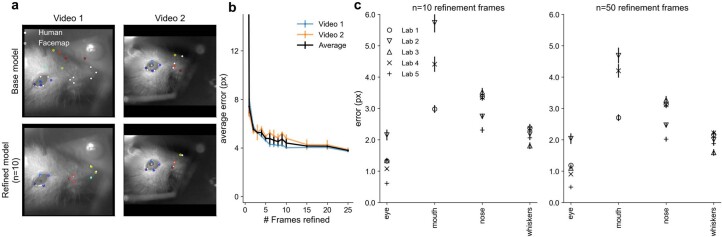
Performance of fine-tuned model on freely-moving mice test frames and performance across subgroups of keypoints on head-fixed mice data. **a**, Top: keypoint predictions using Facemap tracker’s base model (white circles) and human annotations (colored circles) on freely-moving mice face recorded from a head-mounted camera^38^. Bottom: keypoint predictions from the fine-tuned model trained with 10 refined frames. Keypoints labels shown for visible bodyparts only. **b**, Curve shows performance of the Facemap tracker measured by average error (px) (mean ± s.e.m. across n = 50 test frames) as a function of the number of refined frames used for fine-tuning the base model (number of refined frames = 0 is the base model), for each video in **a** and average test error across videos (black). **c**, Average test error across subgroups of keypoints after fine-tuning the base model with n = 10 and n = 50 refined frames used for fine-tuning. Mean across n = 50 test frames, and error bars represent s.e.m. The lab 5 tracking achieved the lowest overall error primarily due to the lack of mouth keypoints in the camera view and the high fidelity tracking of the nose.

We built the Facemap tracker to perform well on a variety of camera angles and across different mice. While Facemap generalized well on data from similar mice and camera configurations, the tracker had variable performance on videos from other labs (Fig. 2a). We investigated whether a fine-tuning strategy might improve the performance of the tracker further on new data. We annotated a small set of video frames contributed by other labs to fine-tune the neural network individually for each lab. The fine-tuned network showed a dramatic drop in error after training with just one frame. Training with around ten frames led to near-optimal performance (Fig. 2b and Supplementary Video 2). This fine-tuning procedure also worked for face videos from freely-moving mice from another lab: with around ten frames, successful tracking was achieved (Extended Data Fig. 3 and Supplementary Video 3)^38^.

Given the quick improvement in the network performance after fine-tuning, we reasoned this step is necessary for adapting Facemap’s tracker to new data. Therefore, we implemented a ‘human-in-the-loop’ workflow to allow users to easily fine-tune Facemap for their own datasets in our graphical user interface (GUI) for the keypoints (Fig. 2c). In the first step, the existing Facemap network is used to generate keypoint predictions. Next, the user refines the predicted keypoints to generate new training labels for the network. Then, the network is re-trained with the new labels to create a fine-tuned network. The fine-tuned network is then applied to new frames and the user can decide whether or not to repeat the retraining process depending on the performance of the fine-tuned network. Once a well-performing network is obtained, the fine-tuned network is saved in the GUI for future use. This fine-tuning step was also used for our experiments in the next section, where we combined keypoint tracking and large-scale neural recordings.

### A deep network model of neural activity

**Fig. 3:**
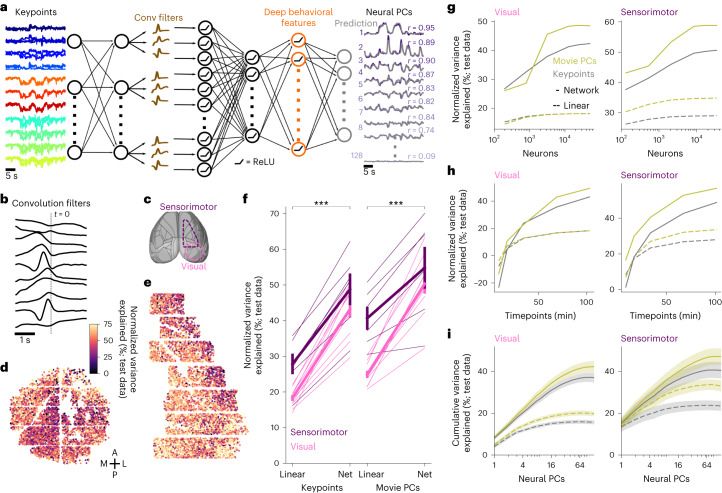
High-accuracy prediction of neural activity using keypoints. **a**, Architecture of five-layer neural network for predicting neural activity. **b**, The resulting temporal convolution filters in layer 2 of the model for an example recording. **c**, Neural recording locations overlaid on the atlas from the Allen Institute for Brain Science (http://atlas.brain-map.org/). **d**, Neurons from an example recording in visual cortex colored by the percentage of normalized variance explained by the deep keypoint model on test data. **e**, Same as **d** for a recording in sensorimotor cortex. **f**, Percentage of normalized variance explained by movie PCs and keypoints, averaged across neurons for each recording. Thick lines denote average across recordings, error bars represent s.e.m., *n* = 16 recordings. Two-sided Wilcoxon signed-rank test, ***P < 0.001 for visual areas (*P* = 3.05 × 10^−5^) and for sensorimotor areas (*P* = 3.05 × 10^−5^). **g**, Same as **f** as a function of the number of neurons, averaged across recordings from visual (left) and sensorimotor (right) areas. **h**, Same as **g**, for the number of timepoints in the training data. **i**, Cumulative variance explained across neural PCs from the keypoints and movie PC predictions. Mean computed across *n* = 16 recordings in 12 mice, error bars represent s.e.m.

**Extended Data Fig. 4:**
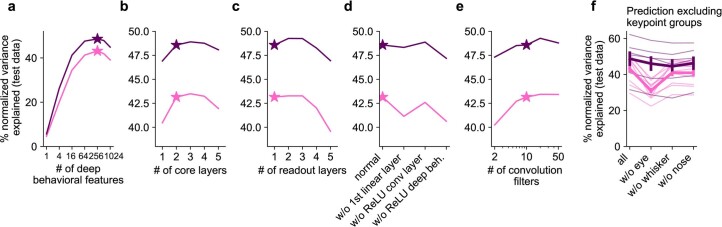
Investigating the network prediction model performance from keypoints. **a**–**e**, We varied different components of the deep network model from keypoints and computed the normalized variance explained across neurons, choosing the architecture denoted with the star. Pink represents the average across visual recordings (n = 10 recordings, 7 mice), and purple represents the average across sensorimotor recordings (n = 6 recordings, 5 mice). **a**, Varying the number of units in the deep behavioral features layer—the last fully-connected layer before the output layer. Star denotes 256 units. **b**, Varying the number of core layers—the layers before and including the deep behavioral features layer, star denotes 2 layers. **c**, Varying the number of readout layers—the layers after the deep behavioral features layer, star denotes 1 layer. **d**, The performance when removing the first linear layer in the network, removing the ReLU non-linearity in the convolution layer, or removing the ReLU non-linearity in the deep behavioral feature layer. **e**, Varying the number of one-dimensional convolution filters, star denotes 10. **f**, Prediction from all keypoints using network, or from all keypoints excluding each face region: eye, whisker and nose. Error bars represent s.e.m.: in visual areas, n = 10 recordings in 7 mice; and in sensorimotor areas, n = 6 recordings in 5 mice.

To determine how neural activity depends on orofacial behaviors, we designed a neural network encoding model that can extract deep features from the keypoint data or directly from the PCs of the videos. Similar to deep encoding models in sensory neuroscience, the model has a first linear step for applying spatiotemporal filters to the time-varying keypoints (Fig. 3a,b), followed by several fully-connected layers that process these features further into more abstract representations that can better predict the neural activity. This deep network model was trained end-to-end to predict the activity of the top 128 PCs of neural data from either visual or sensorimotor cortices at the temporal resolution of the imaging data (300 ms bins; Fig. 3c). The neural activity was split into training and test data in blocks of around 10 min and 3.5 min, respectively, and the variance explained was computed on the test data periods. We normalized the variance explained by an approximate upper bound, estimated using peer prediction, similar to ref. ^14^. Among multiple variations of the neural network architecture, the model we chose (Fig. 3a) had the best performance while using the fewest number of layers (Extended Data Fig. 4).

We found that neurons across visual and sensorimotor cortices were well explained by behavior, with an average normalized variance explained of 43.2% and 48.8%, respectively, from the keypoint-based deep prediction model (Fig. 3d,e). This was computed from the raw variance explained of 4.1% and 5.3% normalized by the explainable variances of 9.4% and 11.1% for the visual and sensorimotor areas, respectively, in bins of ~300 ms.

We compared the deep prediction model to a linear prediction method and found improvements of 136% and 71.5% more explained variance in visual and sensorimotor area, respectively (Fig. 3f). Next, we tested the deep prediction model using as input the PCs of the videos, rather than the keypoints. The deep prediction model again outperformed the linear method with improvements of 102% and 46.2% in visual and sensorimotor area, respectively. The deep model based on movie PCs outperformed the deep model based on keypoints in visual areas (50.4% versus 43.2%) and in sensorimotor areas (54.8% versus 48.8%) (Fig. 3f). This may be due to the much larger number of inputs (500 movie PCs versus 22 keypoint coordinates). Users thus have two options as follows: either use the more interpretable prediction model based on a small number of keypoints or the slightly better performing but less interpretable model based on movie PCs. We also asked how the neural prediction varies with the number of neurons and timepoints available for the model fitting (Fig. 3g,h). We found that explained variance saturated at around 10,000 neurons, but did not saturate with the number of timepoints even for our longest recordings of ~2 h. Thus, large-scale and longer recordings are necessary to fit good encoding models.

**Extended Data Fig. 5:**
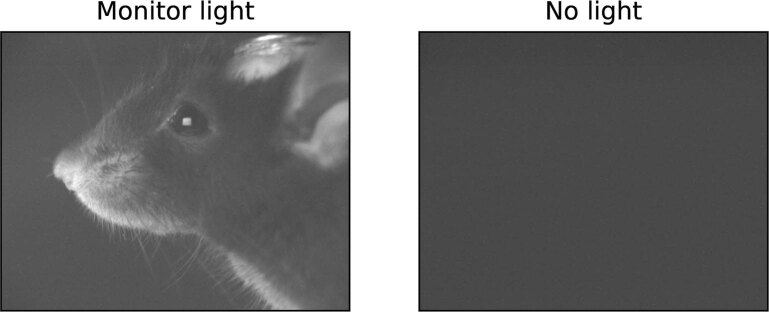
Frame from face videos in various light conditions. We captured videos of the mouse face at various light levels while the infrared LED was turned off and the infrared filter was removed from the camera. Left: the enclosure was closed, but the visual stimulus monitors were on, illuminance of 7.80 lux (experimental setup as in ref. ^64^). Right: the enclosure was closed, and the monitors were off, illuminance of 0.00 lux (experimental setup in this study).

Next, we investigated how the model explained variance changed as a function of neural PC number (Fig. 3i). The largest neural PC generally accounts for the overall arousal state of the mouse, while the higher neural PCs may account for finer and more specific behaviors such as whisking, sniffing and eye movements^14^. In sensorimotor areas, we found that the improvement in variance explained by the neural network model was exclusive to the higher PCs, while the first PC was explained nearly as well (15.1% versus 13.5% for nonlinear versus linear keypoint-based models). In contrast, the first PC of the visual areas benefited substantially from nonlinear prediction (8.1% versus 4.1%). Furthermore, the top PC corresponded to a smaller fraction of the total explained variance in visual compared to sensorimotor areas (ratio = 0.22 and 0.37 of explained variance at 1 versus 64 neural PCs in Fig. 3i). Overall, these differences suggest that the behavior-related neural activity in visual areas is higher-dimensional and more nonlinear as a function of behavior compared to sensorimotor areas. The differences cannot be explained by visual inputs, because the recordings were performed in complete darkness with measured lux values of 0.00 in the visible spectrum (for comparison, we obtained 7.8 lux with monitors on and 84.4 lux with the microscope doors open; see also Extended Data Fig. 5).

**Fig. 4:**
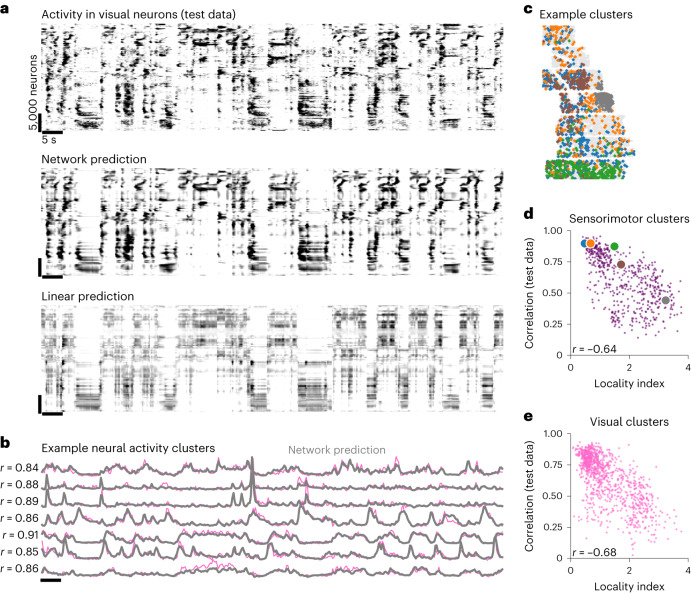
The deep network model predicts fine features of neural activity. **a**, Top, neurons from a visual (posterior, dorsal cortex) recording during spontaneous behavior. Each row represents averaged activity of 25 neurons, sorted using Rastermap^46^. The time period is during a held-out test period. Middle, predicted neural activity from the deep network model fit using keypoints in Fig. 3a. Bottom, predicted neural activity using linear prediction from the keypoints. **b**, Example neural activity clusters, same time period as **a**. **c**, Spatial locations of neurons from five example clusters from a recording in sensorimotor cortex. **d**, The locality index of each sensorimotor cluster across recordings, defined by the KL divergence between its spatial distribution and the distribution of all the neurons, plotted against the correlation of the cluster activity with its prediction from the deep network model. The colored circles correspond to the clusters in **d**. **e**, Same as **d**, for visual clusters.

**Extended Data Fig. 6:**
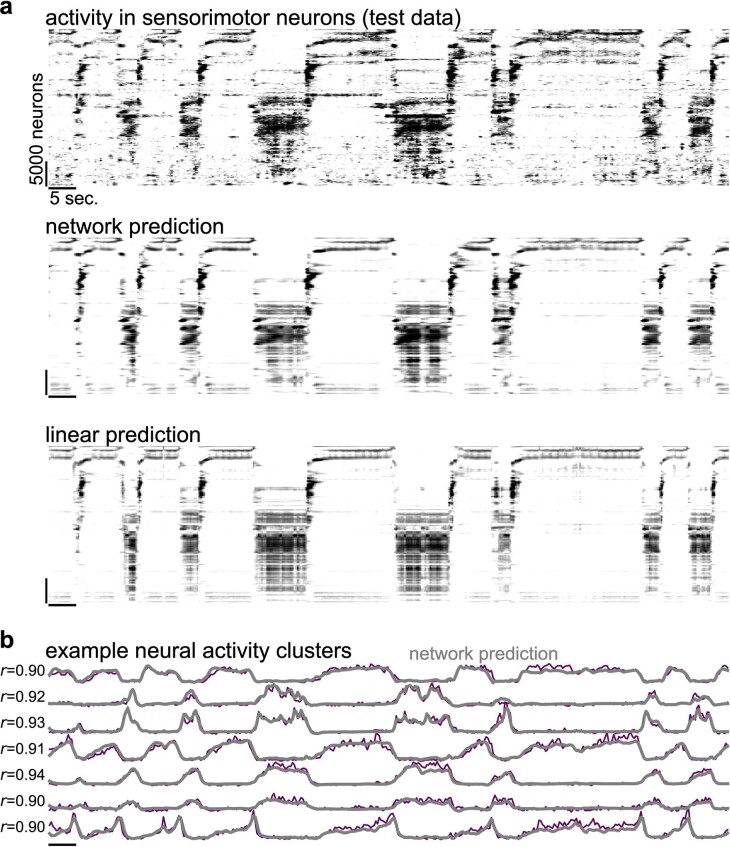
Activity in an example sensorimotor recording. **a**, Activity of the sensorimotor recording during a test period shown in Fig. 3e and Fig. 4c, sorted by Rastermap, along with neural predictions from the deep network model and the linear model from the keypoints. **b**, Example neural activity clusters from the recording (purple), plotted with the prediction from keypoints in gray.

**Extended Data Fig. 7:**
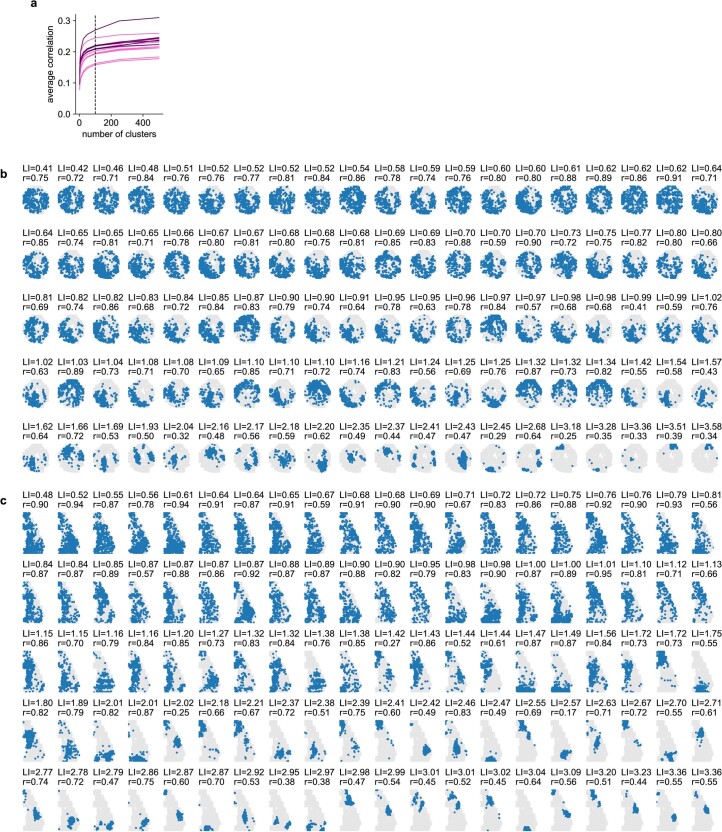
Neural activity clusters from a visual and a sensorimotor recording. **a**, Correlation of each neuron with its assigned cluster center across time, averaged across all neurons per recording, as a function of the number of cluster centers used in the clustering algorithm (n = 16 recordings from 12 mice). **b**, The spatial locations of neurons from each neural activity cluster from the recording shown in Fig. 3d and Fig. 4a,b. Blue indicates neurons in the cluster, and gray indicates all other neurons. LI = locality index, r = correlation with behavior prediction on test data. **c**, Same as b, for the neural activity clusters from the sensorimotor recording shown in Fig. 3d, Fig. 4c and Extended Data Fig. 6.

The nonlinear, deep network model predicted the fine structure of neural activity, capturing small events across small groups of neurons better than the linear model (Fig. 4a and Extended Data Fig. 6). We investigated the spatial distribution of these subgroups of neurons by clustering the neurons with *k*-means into 100 clusters, a number that was sufficient to achieve a high correlation of each neuron with its cluster center (Extended Data Fig. 7a and Fig. 4b,c). Some clusters were spatially spread throughout the recording area, while others were more localized (Fig. 4c and Extended Data Fig. 7b,c). We defined a spatial locality index for each cluster (Methods). In general, the clusters best predicted by the behavior had the lowest locality index (Fig. 4d,e). Thus, behaviorally-correlated clusters are more spatially distributed across cortex, consistent with the hypothesis that many of these behavioral signals are broadcast across the brain.

### State dynamics extracted from deep behavioral features

The last hidden layer in the deep network model, the ‘deep behavioral features’, contains a representation of behavior that is not directly available in the raw keypoints. To understand the nature of these representations, we characterized their dynamical properties using HMMs. Various types of HMMs have been previously fit to raw behavioral data, often from freely-moving animals^21,39–42^. Here we chose to use discrete HMMs, which can model the data as a succession of discrete states^43,44^. Transitions between states are probabilistic with probabilities defined by the transition matrix. In addition, each state is assigned a fixed ‘emission’ pattern of activations across all features. The transition matrix and emission patterns are parameters that are fit to each session individually.

**Fig. 5:**
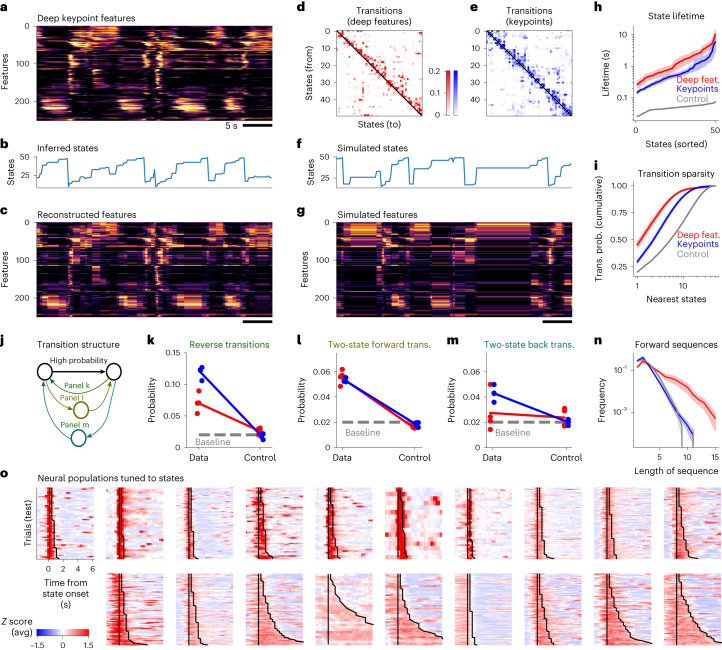
The deep behavioral features have highly-structured dynamics. **a**, Example dynamics of deep behavioral features computed by the neural network in Fig. 3a. The features have been sorted along the *y* axis using a one-dimensional t-SNE embedding^45^. **b**, Inferred states using an HMM. **c**, Reconstructed features using the inferred states on a test trial. **d**, State transition matrix of the HMM. Self-transitions were set to 0 and the rows were renormalized to 1. States have been sorted to maximize the sum of transition probabilities above the diagonal, using the Rastermap algorithm^46^. **e**, Same as **d** for HMMs fit directly to the keypoint data. **f**, Simulated states using the HMM fit to the deep behavioral features. **g**, Simulated features from the HMM. **h**, Distribution of inferred state lifetimes using the self-transition probabilities of the HMM, averaged across *n* = 5 recordings from five mice, and error bars represent s.e.m. See Methods for description of controls for all panels. **i**, Probability of transitions to *n*-nearest states as a function of *n*. The average is taken over all initial states, and the line represents the average across *n* = 5 recordings from five mice. **j**, Schematic for **k**–**m**. For each pair of states with a high transition probability, certain other transition probabilities are reported. Each dot represents transitions from a different animal, averaged across all high-probability pairs. **k**, Average probability of reverse transitions. Baseline is computed as the average transition probability across all state transitions. **l**, Probability of two-state transitions. **m**, Probability of two-state backward transitions. **n**, Distribution of ‘forward’ sequence lengths, where the forward direction is defined as higher indices in the Rastermap sorting of states from **d**, averaged across *n* = 5 recordings from five mice, and error bars represent s.e.m. **o**, Neural populations tuned to 19 selected states (of 50 total). The top 300 most selective neurons were chosen on train trials, and their average on test trials is shown. Vertical lines indicate trial onsets, while the second jagged line indicates trial offsets.

**Extended Data Fig. 8:**
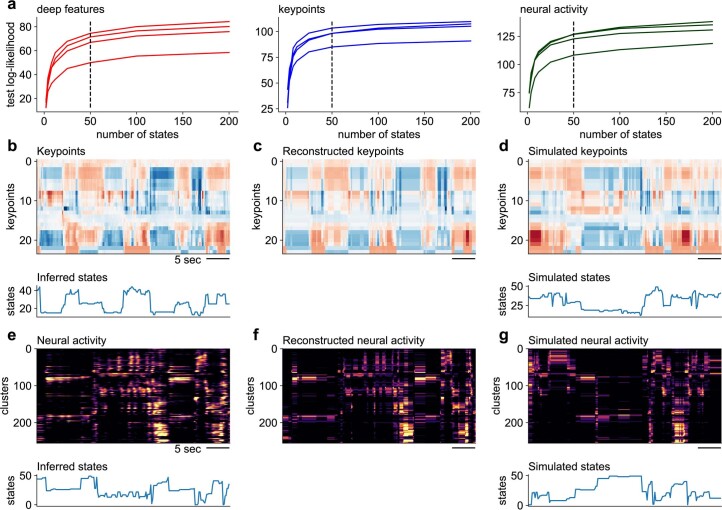
HMM modeling of deep features, keypoints and neural activity. **a**–**c**, Test log-likelihood for HMM models trained on deep features (a), keypoints (b) and neural activity clusters (c), as a function of the number of hidden states in the HMM. Curves represent different mice. **b**, Top: visualization of keypoint data (after *z* scoring). Bottom: inferred states by the HMM model. **c**, Reconstructed keypoint data from the inferred HMM states. **d**, Top: simulated keypoint data from the HMM. Bottom: simulated state dynamics. **e**–**g**, Same as b–d but for modeling neural activity clusters. Each cluster represents the average activity of 100–300 neurons.

We start by visualizing the HMMs that were fit to an individual session using 50 states, which were sufficient to reach a high log-likelihood on test trials (Extended Data Fig. 8a). The 256 deep behavioral features from one session were first sorted using one-dimensional t-SNE, such that features with similar activation patterns are near each other in the plot (Fig. 5a)^45^. The most probable states can then be inferred (Fig. 5b), and their emission patterns can be used to reconstruct the original data matrix (Fig. 5c). The reconstruction assures us that the HMM captures a majority of the data variance. We also visualized instances of the same state and observed they were consistent and in some cases human-interpretable (Supplementary Videos 4). The HMM states were also separately sorted using Rastermap^46^, so that forward transitions—from a lower to a higher state in the sorting—are maximized in the sorting (Fig. 5d). Due to this sorting, state dynamics appear to be arranged in ordered, increasing sequences (Fig. 5b). This asymmetry in state transitions was not apparent at the level of the keypoints themselves (Fig. 5e and Extended Data Fig. 8b–d); despite being sorted with the same Rastermap algorithm, states inferred directly from keypoints had relatively symmetric transition probabilities. To further validate the quality of the HMM, we used it to generate new synthetic data (Fig. 5f,g). Samples from the model had the same overall appearance as the original data. Thus, transition probabilities captured in the HMM can generate the same kind of behavioral sequences as are present in the data itself.

Next, we quantified some of the HMM properties directly. The duration of a state in the model is given by the self-transition probability (which was left out from the visualizations in Fig. 5d,e). Self-transitions *p* near 1, imply a long-lasting state, with an exponential distribution of state durations. The mean of this distribution is defined as the ‘state lifetime’, and can be easily computed as −log(1 − *p*). The distribution of state lifetimes was broad (Fig. 5h), with lifetimes ranging from 0.2 to 10 s. The model fit to behavioral states had longer lifetimes than the model fit directly to the keypoints, and both had much longer lifetimes than a control model fit to temporally-shuffled data. For the rest of our analyses, we will ignore self-transition probabilities and will focus on the transition probabilities between states. Operationally, we set self-transitions to 0 in the transition matrix and normalize the outgoing transitions to a sum of 1 (like in Fig. 5d,e).

Another property of the HMM is the sparsity of transitions between states. It is apparent in Fig. 5d that the transition matrix is quite sparse, with most values near-zero and a few large values. In other words, HMM states tend to transition to only a few other potential states. To quantify this property, we computed for each state the probability to transition to its *n*-nearest states, where near states are defined as the ones with the highest probability of transition. As *n* increases, the summed transition probability approaches its maximum of 1 quickly for small *n* and much more slowly after. This shows that the HMM has a sparse structure, dominated by a few large transitions. In contrast, models inferred from the keypoints had more dense transitions (approached 1 more slowly). Both types of models had sparser transitions than the control model that was fit to appropriately-shuffled data (Methods).

To quantify the asymmetry of the HMM transitions, we performed a series of analyses directly on the transition matrix (Fig. 5j). For each pair of states with high transition probability, we asked how likely other transitions are. We analyzed reverse transitions (Fig. 5k), two-step forward transitions (Fig. 5l) and two-step backward transitions (Fig. 5m). We found that these types of transitions were generally more likely than chance. However, reverse transitions were less likely in the deep feature HMM compared to the keypoint HMM, corresponding to the more asymmetrical nature of the former model (Fig. 5d versus Fig. 5e). While two-step forward transitions were matched between the two models, the two-step backward transitions were at baseline levels for the deep feature HMM, but not for the keypoint HMM. The net effect of the asymmetry in state transitions was that the deep feature HMM produced longer, uninterrupted forward sequences of states. We quantified this property from the inferred states, measuring the length of all increasing state sequences (Fig. 5n). The distribution of forward sequence lengths was much more long-tailed for the deep feature HMM, compared to controls and to the keypoint HMM (Fig. 5n). Combined with the already longer state durations (Fig. 5h), this shows that the deep behavioral features have longer, uninterrupted runs of stereotypical dynamics. This may imply that the HMM states inferred from deep behavioral features correspond to more abstract aspects of behavior, which may be ignoring some specific low-level properties of the keypoints such as the phases of the whisking, sniffing or running cycles. Furthermore, the asymmetrical transitions may correspond to a much longer cycle of behavior dictated by transitions between passive to active states and back. More work will be needed to fully make this link, perhaps using more sophisticated HMMs such as switching linear dynamical systems^21^.

### Relation between deep behavioral dynamics and neural dynamics

**Extended Data Fig. 9:**
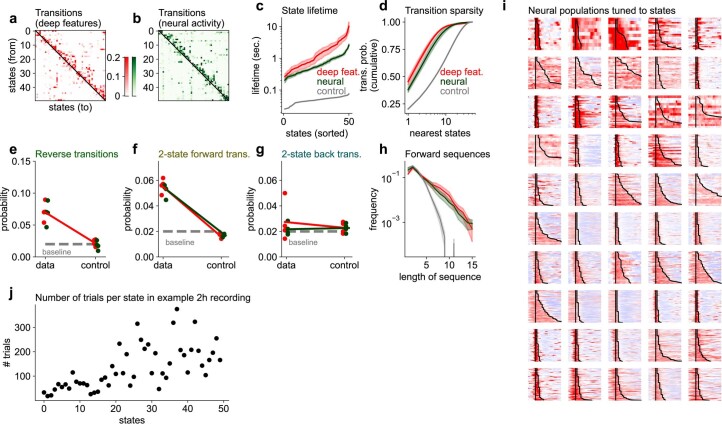
Comparison of HMM models of deep features and neural activity. **a**,**b**, Example transition matrices for HMM models with 50 states trained on the deep features (**a**) or the neural activity (**b**). **c**, Distribution of state lifetimes, and error bars represent SEM. **d**, Transition matrix sparsity quantified as cumulative transition probability from one state to all other states, and error bars represent SEM. **e**–**g**, Reverse, 2-state forward and 2-state backward transitions. **h**, Distribution of forward sequence lengths, and error bars represent SEM. **i**, Neural population responses to all 50 states for the recording illustrated in Fig. 5o. **j**, Number of trials per state for the recording illustrated in Fig. 5o.

To directly compare the behavioral HMM to the neural data, we visualized the activity of the neural populations tuned to different HMM states. We define a ‘trial’ as uninterrupted timepoints of the same state, and the response of a neuron on that trial as its average activity over those timepoints. Across states, we observed a range of approximately 50–300 trials per state (Extended Data Fig. 9j). We then used training trials to select the neurons with the highest activity on each state. For many of the states, we obtained neural populations highly selective to that state (see Fig. 5o for a subset of states and Extended Data Fig. 9i for all 50 states). We observed populations with either brief or long-lasting activity, which mirrored the diversity of behavioral state durations. We note that the existence of these neural populations does not follow directly from the fitting procedure of the deep features; while the deep features were indeed trained to predict neural activity, we allowed arbitrary weight combinations of these features to predict single neurons as opposed to relating single neurons to discretized behavioral states as we do in this section. Other aspects of these neural populations could be investigated further, for example, by engaging these neural populations in a behavioral task and comparing their activity with the deep behavioral features they represent. However, that is beyond the scope of the present study.

We have so far used HMMs to study changes in dynamical properties which are a consequence of the deterministic transformation from keypoints to deep behavioral features. We can also compare the dynamical properties of deep behavioral features to those of the neural data itself. To do this, we first clustered the recordings of ~50,000 neurons into 256 clusters (chosen to match the number of deep behavioral features) using *k*-means and fit the HMMs to the mean cluster activities. The neural HMM had relatively shorter state durations and more dense state transitions (Extended Data Fig. 9a–d), but similarly asymmetric transition probabilities to the deep behavioral HMM (Extended Data Fig. 9e–h). Visualizing the neural data and the inferred neural HMM states (Extended Data Fig. 8e–g), we can see that neural activity contains some shorter states with faster transitions compared to the deep behavioral features (Fig. 5a,b). We conclude that the deep behavioral features especially capture longer-duration states in the neural data and may be missing information about the shorter-duration states.

## Discussion

Here we described Facemap, a framework that relates orofacial tracking to neural activity using new modeling tools. The framework is composed of two parts as follows: (1) an orofacial keypoint tracker for extracting eye, whisker, nose and mouth movements, and (2) a neural network encoding model that extracts spatiotemporal features of behavior that are most related to the neural activity. We have shown that the orofacial tracker is highly accurate while being substantially faster than other keypoint tracking approaches, and we showed that it can be easily trained on new orofacial videos from other experimental setups than our own. These keypoints capture the important aspects of the behavior with many fewer variables (22) than the number of pixels in a frame (~100,000). Despite this dramatic dimensionality reduction, the keypoints contain substantial information about the behavior to predict neural activity very accurately.

We used the new Facemap framework to make a few initial observations. We found that the eye keypoints had predictable dynamics on much longer timescales (10 s) compared to the dynamics of the nose keypoints (1 s), while the whisker dynamics were somewhere in-between. Across both visual and sensorimotor areas, clusters that were spread out over the brain were the ones best predicted from behavior. We also found that visual cortex has higher-dimensional and more nonlinear representations of behavior compared to sensorimotor cortex, a surprising result that merits further investigation.

We also found that the deep behavioral features extracted by the network model contained a much more orderly representation of behavior compared to the raw keypoints. Using an HMM, we found that the deep behavioral features were organized into relatively longer-lasting states, from less than a second to several seconds, which transitioned into other states in a predictable manner, forming sequences of states that repeated many times over the course of a session. These asymmetric state sequences were not found in the raw keypoints and had substantially longer durations in the deep behavioral features compared to the neural activity. The differences arise because the deep features represent specifically those aspects of the behavior that best predict the neural data. There are important aspects of behavior that may not be relevant for this prediction, such as the phases of the whisking, sniffing or running cycles, and there are aspects of the neural activity that may not be predictable at all. When those are factored out, an orderly representation emerges in the deep behavioral features.

These initial analyses are just the start of using Facemap to extract insights about neural activity patterns and the structure of behavior itself. We developed the method alongside a user-friendly GUI so that others can easily adapt it to their own data, and use it flexibly in their own studies. To track fast orofacial movements such as whisker movements, we note that reasonable resolution of the face will likely be required (at least 200 pixels) and a frame rate of at least 50 Hz. Many labs already have video cameras capturing the face of the mouse with sufficient resolution and frame rate and could, therefore, perform orofacial tracking during such experiments^47,48^. Furthermore, with head-mounted cameras^38^, orofacial tracking can be incorporated into freely-moving behavioral contexts, to enable observation of the fine movements that rodents make as they explore their environment or engage in social interactions^39,49–53^. We believe Facemap is one of the important steps toward unlocking the fundamental mystery of brain-wide neural activity—what is its function and where is it coming from—and we look forward to seeing it used to make progress on these questions.

## Methods

All experimental procedures were conducted according to IACUC and received ethical approval from the IACUC board at HHMI Janelia Research Campus. The Facemap code library is implemented in Python 3 (ref. ^54^), using pytorch, numpy, scipy, numba, tqdm, opencv and pandas^34,55–59^. The GUI additionally uses PyQt and pyqtgraph^60,61^. The figures were made using matplotlib and jupyter-notebook^62,63^.

### Data acquisition

#### Animals

We performed 16 recordings in 12 mice bred to express GCaMP6s in excitatory neurons: TetO-GCaMP6s x Emx1-IRES-Cre mice (available as RRID:IMSR_JAX:024742 and RRID:IMSR_JAX:005628). These mice were male and female and ranged from 2 to 12 months of age. Mice were housed in reverse light cycle and were pair-housed with their siblings before and after surgery. Due to the stability of the cranial window surgery, we often use the same mice for multiple experiments in the lab; five of the seven visual area mice were used in a previous study^64^, and the other two visual mice and all of the sensorimotor mice were trained on behavioral tasks after the recordings.

#### Surgical procedures

Surgeries were performed in adult mice (P35–P125) following procedures outlined in ref. ^64^. In brief, mice were anesthetized with isoflurane, while a craniotomy was performed. Marcaine (no more than 8 mg kg^−1^) was injected subcutaneously beneath the incision area, and warmed fluids + 5% dextrose and buprenorphine 0.1 mg kg^−1^ (systemic analgesic) were administered subcutaneously along with dexamethasone 2 mg kg^−1^ via intramuscular route. For the visual cortical windows, measurements were taken to determine bregma–lambda distance and location of a 4 mm circular window over V1 cortex, as far lateral and caudal as possible without compromising the stability of the implant. A 4 + 5 mm double window was placed into the craniotomy so that the 4 mm window replaced the previously removed bone piece and the 5 mm window lay over the edge of the bone. The sensorimotor window was also a double window and it was placed as medial and frontal as possible. The outer window was 7 mm × 4.5 mm and the inner window was around 1 mm smaller in all dimensions. After surgery, Ketoprofen 5 mg kg^−1^ was administered subcutaneously and the animal was allowed to recover on heat. The mice were monitored for pain or distress, and ketoprofen 5 mg kg^−1^ was administered for 2 days following surgery.

#### Videography

The camera setup was similar to the setup in ref. ^14^. A Thorlabs M850L3 (850 nm) infrared LED was pointed at the face of the mouse to enable infrared video acquisition in darkness. The videos were acquired at 50 Hz using FLIR cameras with a zoom lens and an infrared filter (850 nm and 50 nm cutoff). The camera acquisition software was BIAS (https://github.com/janelia-idf/bias). The wavelength of 850 nm was chosen to avoid the 970 nm wavelength of the two-photon laser while remaining outside the visual detection range of the mice^65,66^.

The entire setup was enclosed in a large black box to prevent light from the room from entering the microscope light path and from entering the mouse’s eye. We turned off the infrared LEDs and then estimated the amount of visible non-infrared light entering the mouse’s eye during recording by using an FLIR Extech LT300 Light Meter. We positioned the Light Meter where the mouse’s head is during recording. We found that when the enclosure was closed, as in our experimental conditions, the illuminance measurement was 0.00 lux. When we kept the enclosure closed but turned on the monitors to show visual stimuli (as in ref. ^64^), the illuminance measurement was 7.80 lux. We captured the face of the mouse with our camera in these two settings, with the infrared filter removed from the camera (Extended Data Fig. 5). For comparison, the illuminance of the enclosure area when it was open, coming from overhead lighting in the room, was much greater at 84.4 lux.

#### Imaging acquisition

We used a custom-built two-photon mesoscope^67^ to record neural activity, and ScanImage^68^ for data acquisition. We used a custom online *Z*-correction module (now in ScanImage) to correct for *z* and *x-y* drift online during the recording. As described in ref. ^64^, we used an upgrade of the mesoscope that allowed us to approximately double the number of recorded neurons using temporal multiplexing^69^.

The mice were free to run on an air-floating ball. Mice were acclimatized to running on the ball for several sessions before imaging. On the first day of recording, the field of view was selected such that large numbers of neurons could be observed, with clear calcium transients.

#### Processing of calcium imaging data

Calcium imaging data were processed using the Suite2p toolbox^70^ (available at www.github.com/MouseLand/suite2p), which relies on the packages numpy, scipy, numba, scanimage-tiff-reader, paramiko and scikit-learn^57,71–74^. Suite2p performs motion correction, ROI detection, cell classification, neuropil correction and spike deconvolution as described elsewhere^14^. For non-negative deconvolution, we used a timescale of decay of 1.25 s (refs. ^75,76^). We obtained 50,614 ± 13,919 (s.d., *n* = 10 recordings) neurons in the visual area recordings, and 33,686 ± 4,465 neurons (*n* = 6 recordings) in the sensorimotor area recordings.

### Facemap tracker network

#### Model architecture

The Facemap tracker network is a U-Net-style convolutional neural network consisting of downsampling and upsampling blocks with skip connections implemented in pytorch^34^. The model’s input is a grayscale 256 × 256 pixels image, which is passed through a set of convolutional filters of different sizes, as shown in Fig. 1b. The network has two sets of outputs as follows: (1) heatmaps represent the probability of a keypoint in the pixel region and (2) location refinement maps represent the *x* and *y* offsets between the keypoint position in full-sized image and the downsampled map, similar to refs. ^27,33^. The downsampled (64 × 64 pixels) heatmaps and location refinement maps are used to obtain the *x* and *y* coordinates of keypoints, and example traces are shown in Fig. 1f.

The tracker predicted 15 distinct keypoints in total for tracking mouse orofacial movements from different views (Fig. 1a and Extended Data Fig. 1). The keypoints were used to track various movements of the eye (4), nose (5), whiskers (3), mouth (2) and an additional keypoint for the paw. The forepaw occasionally entered the view, such as during grooming, but we found this keypoint difficult to track and use in further analyses, so we did not consider it further. We also labeled a fifth nose keypoint (nose bridge, not shown in Fig. 1a and Extended Data Fig. 1), but found that it was difficult to identify across different camera angles and, therefore, excluded it from the analyses in the article. The videos taken during neural recordings were from the view in Fig. 1c. In this view, the mouth keypoints were not visible, so those keypoints were not used in the model for neural prediction. Thus, we used four eye keypoints, four nose keypoints and three whisker keypoints for neural prediction.

#### Training

The Facemap tracker was trained on 2,400 images recorded from multiple mice and different camera views (Extended Data Fig. 1). Training images of size 256 × 256 pixels were labeled with all the keypoints, except when a bodypart was not visible in the frame, then no label was added. The model was trained for 36 epochs with the Adam optimizer using a batch size of 8 and weight decay of zero^77^. We used a custom learning rate (LR) scheduler that used a fixed LR of 0.0004 for 30 epochs followed by $\frac{1}{10}{\rm{LR}}$ for the next three epochs and finally $\frac{1}{25}{\rm{LR}}$ for the final three epochs. Each image was normalized such that 0.0 represented the first percentile and 1.0 represented the 99th percentile. Image augmentations performed during training were random crop, resize after padding to maintain aspect ratio, horizontal flip and contrast augmentation.

#### Performance evaluation

The accuracy of the tracker was evaluated using the average pixel error for 100 test frames of size 256 × 256 pixels from a new mouse and different camera views. First, the Euclidean distance in pixels between the ground-truth labels and the predicted keypoints was computed. Next, the average error was computed as the average of the Euclidean distances across all frames (Extended Data Fig. 2a) and all keypoints (Fig. 1e).

The processing speed of the tracker was calculated to evaluate its utility for offline and online analyses. Therefore, the processing speed calculation accounted for the timing of various steps as follows: (1) image preprocessing, (2) forward pass through the model and (3) postprocessing steps. All processing speeds are reported for a sample image of size 256 × 256 pixels passed through the network for 1,024 repetitions and a total of ten runs using various batch sizes on different GPUs (Supplementary Table 2).

#### Filtering keypoint traces for neural prediction

Occasionally, keypoints are occluded, such as during grooming. Therefore, like DeepLabCut, we found the timepoints when the tracker network confidence was low, and replaced those timepoints in the keypoint traces by a median-filtered value. The network confidence, or likelihood, is defined as the value of the peak of the heatmap output. The likelihood traces for each keypoint were baseline filtered in time with a Gaussian filter of s.d. of 4 s, then the threshold of the likelihood was defined as negative eight times the s.d. of the baselined likelihood, and any values below this threshold were considered outliers. This identified on average 0.19% of timepoints across all keypoint traces as outliers.

After excluding outliers based on likelihood, we also directly identified outlier timepoints using the keypoint traces, by detecting large movements or deviations from baseline. If a keypoint moved more than 25 pixels from the previous timepoint to the current timepoint, then the current timepoint was considered an outlier. Also if the keypoint trace on the current timepoint exceeded its median-filtered (window = 1 s) value by more than 25 pixels, then the current timepoint was considered an outlier. This identified on average an additional 0.066% timepoints across all keypoint traces as outliers.

To obtain values for the outlier timepoints, we median-filtered the keypoint traces with a window of 300 ms, excluding the outlier timepoints. Linear interpolation from the median-filtered traces was then used to fill in the values at the outlier timepoints.

### Pose estimation model comparisons

We compared the performance of the Facemap tracker to other state-of-the-art tools used for pose estimation, including SLEAP^31^ and DeepLabCut^27,36^. The models were trained on the same training set used for Facemap. In addition, the same protocol for speed benchmarking was used to obtain the processing speed of the other models.

#### DeepLabCut models training

DeepLabCut’s models used for comparison included two different architectures as follows: ResNet50 (default model) and Mobilenet_v2_0.35 (fastest model). Augmentations used during training were scaling, rotation and contrast augmentation, similar to training of the Facemap tracker. A hyperparameter search was performed to find optimal training parameters for each model using different batch sizes (1, 2, 4 and 8) and LRs (0.0001, 0.001 and 0.01). Models with the lowest average test error for each architecture were compared to Facemap in Fig. 1e and Extended Data Fig. 2.

Processing speeds for DeepLabCut’s models were obtained using a similar approach as Facemap tracker. We timed DeepLabCut’s getposeNP function for 1,024 repetitions for a total of ten runs for different batch sizes and GPUs. The getposeNP function timing included a forward pass through the network and postprocessing steps to obtain keypoints locations from the heatmaps and location refinement maps.

#### SLEAP models training

The default U-Net backbone was used for SLEAP’s models, which included the following two different values of initial number of filters: (1) *c* = 16 (default) and (2) *c* = 32 to vary the network size and potentially improve accuracy. A hyperparameter search over different LRs (0.0001, 0.001 and 0.01), batch sizes (1, 2, 4 and 8) and number of epochs (100 and 150) was performed to find the best model for each U-Net configuration. Furthermore, early stopping by stopping training on plateau was used for half of the models to prevent overfitting. Default augmentation settings were used for most models and mirroring (horizontal flip) was added to some models to match the training of the other networks used for comparison. Similar to DeepLabCut, the best models were selected based on the lowest average test error for the default and *c* = 32 models and used in Fig. 1e and Extended Data Fig. 2.

The processing speed for SLEAP’s models was calculated by timing their predict_on_batch function. The U-Net models with different numbers of initial filters were run for 1,024 repetitions for a total of ten runs using different batch sizes of our sample image input.

### Facemap tracker refinement

We developed a method for fine-tuning the Facemap tracker for new data that differed from our training data. Facemap tracker’s base model is defined as the network trained on our dataset (Fig. 1). We extracted frames from videos contributed by five other labs to use as training data for fine-tuning the base model specifically to each lab’s video. We executed the following steps for each lab’s video. First, the base model was used to generate predictions for 50 random frames. Keypoints on the 50 training frames were refined to correct keypoints with large deviations from their defined bodyparts or remove keypoints not in view. The percentage of keypoints refined across 50 frames were 99.51% for lab 1, 100% for lab 2, 99.79% for lab 3, 100% for lab 4 and 98.32% for lab 5. Therefore, most of the keypoints across all frames were refined for fine-tuning the model and benchmarking the fine-tuned model.

Next, the base model was fine-tuned with varying numbers of training frames ranging from 1 to 50. The network was trained for 36 epochs with an initial LR of 0.0001 with annealing as described earlier and a weight decay of 0.001. Additionally, we trained a model from scratch, that is a network initialized with random weights, using ten training frames for comparison. To compute the errors for the base model, the fine-tuned model and the scratch model, we used 50 test frames and labeled them from scratch to use as a test set. We then computed the average error in pixels from the test set labels to the model predictions (Fig. 2b). The models trained from scratch with ten frames had an average error of 3.76 ± 0.39 pixels across labs, compared to 2.43 ± 0.24 pixels for the base model fine-tuned with ten frames. Predictions from the base, scratch and fine-tuned models for a random section of the video are shown in Supplementary Video 2 for each lab. The workflow used for the analysis was integrated into the GUI so users can easily fine-tune the Facemap tracker with video recordings that differ from our training data (Fig. 2c).

### Autoregressive model for prediction of keypoints

We built an autoregressive model to determine how far into the future we could predict each keypoint, as a measure of its timescale. The keypoint traces were split into ten segments in time. The first 75% of each segment was assigned to the training set, and then after 2.6 s which were excluded, the remaining part of the segment was assigned to the test set. Linear regression was performed with exponential decay basis functions, with decay timescales from 40 ms to 5 s. All keypoint traces were input to the basis functions, then combined linearly to predict each future timepoint predicted. We fit the regression model on training timepoints separately for each future timepoint, for timepoints 20 ms to 10 s in intervals of 20 ms and for 10 s to 40 s in intervals of 500 ms. Then we estimated performance on test timepoints at each future timepoint delay using variance explained. We estimated the timescale of the keypoint trace as the future timepoint at which the variance explained was half the variance explained at a time delay of 20 ms.

### Behavior to neural prediction

The activity of each neuron was *z*-scored: the activity was subtracted by the mean and divided by the s.d. To predict the neural activity from behavior, we reduced the dimensionality of the *z*-scored activity using singular value decomposition (SVD) and keeping 128 components, obtaining *U*, *S* and *V* matrices of size (neurons by 128; 128; timepoints by 128, respectively). We then predicted the neural PCs, which we defined here as the product of *V* and *S*, calling this *Y* = *VS*. After obtaining a prediction of the neural PCs $\hat{Y}$, we projected the prediction into the neural activity space using *U*, so that the predicted neural activity was defined as $U{\hat{Y}}^{\top }$. If fewer than 200 neurons were predicted, then we directly predicted the neurons rather than using the PCs. When predicting more neurons, we found that predicting the neural PCs performed and/or outperformed direct neural prediction.

The neural activity was split into ten segments in time. The first 75% of each segment was assigned to the training set, and then after 3 s which were excluded, the remaining part of the segment was assigned to the test set. The training and test sets were made to consist of continuous segments to avoid contamination of the test set with the train set due to the autocorrelation timescale of behavior, with lengths on average of 10 and 3.5 min, respectively.

We quantified the performance of a neural prediction model using the variance explained. The single neuron variance explained for a neural trace for neuron *i* (${\overrightarrow{s}}_{i}$) is defined as

1 $${{{{\rm{VE}}}}}_{i}=1-\frac{{\left({\overrightarrow{s}}_{i}^{{{{\rm{test}}}}}-{\overrightarrow{s}}_{i}^{{{{\rm{pred}}}}}\right)}^{\top }\left({\overrightarrow{s}}_{i}^{{{{\rm{test}}}}}-{\overrightarrow{s}}_{i}^{{{{\rm{pred}}}}}\right)}{{{{\rm{var}}}}\left({\overrightarrow{s}}_{i}^{{{{\rm{test}}}}}\right)},$$

which is the s.d. for variance explained.

#### Peer prediction analysis

Neurons have independent noise that models cannot explain. Therefore, an upper bound for the variance that a model can explain is lower than the total variance of neural activity. To estimate the amount of this explainable variance in the neural recordings, we used the ‘peer prediction’ method^14,78,79^. Peer prediction analysis predicts each neuron from the other simultaneously-recorded cells (the neuron’s ‘peers’). The amount of variance that the peer prediction model explains is an estimate of the repeatable shared variance across neurons, we term this variance the explainable variance.

To compute peer prediction, we split the population into two spatially segregated populations, dividing the field of view into nonoverlapping strips of width 200 μm and assigning the neurons in the even strips to one group, and the neurons in the odd strips to the other group, regardless of the neuron’s depth. Next, we computed the top 128 PCs of each population and predicted one population’s PCs from the other population’s PCs using RRR fit to training data with *λ* = 1 × 10^−^^1^ and rank = 127. The variance explained by this model on test data (Eq. (1)) is termed the explainable variance for each neuron. The average explainable variance was 9.4% in the visual recordings and 11.1% in the sensorimotor recordings at the recording frame rate of 3 Hz.

#### Prediction performance quantification

We computed the variance explained for a given behavioral prediction model for each neuron on test data (Eq. (1)). The average single neuron variance explained, in 300 ms bins, by the deep network model using keypoints was 4.1% in the visual areas and 5.3% in the sensorimotor areas, and using movie PCs was 4.8% and 5.3%, respectively. We then normalized the variance explained by the upper bound on its variance explained, the explainable variance, as computed from peer prediction. We quantified the normalized variance explained on a per-neuron basis in Fig. 3d,e, taking the variance explained for each neuron and dividing it by its explainable variance, and visualizing only the neurons with an explainable variance greater than 1 × 10^−^^3^. For population-level variance explained quantification, the normalized variance explained was defined as the mean variance explained across all neurons divided by the mean explainable variance across all neurons (Fig. 3f–i and Extended Data Fig. 4).

We also computed the cumulative variance explained across neural PCs (${\overrightarrow{y}}_{i}$), defined as

$$\begin{array}{l}{{{{\rm{VE}}}}}_{i,{{{\rm{cumulative}}}}}=\\ \frac{\mathop{\sum }\nolimits_{k = 0}^{i}{{{\rm{var}}}}\left(\;{\overrightarrow{y}}_{k}^{{{{\rm{test}}}}}\right)-{\left(\;{\overrightarrow{y}}_{k}^{{{{\rm{test}}}}}-{\overrightarrow{y}}_{k}^{{{{\rm{pred}}}}}\right)}^{\,\top }\left(\;{\overrightarrow{y}}_{k}^{{{{\rm{test}}}}}-{\overrightarrow{y}}_{k}^{{{{\rm{pred}}}}}\right)}{\mathop{\sum }\nolimits_{k = 0}^{128}{{{\rm{var}}}}\left(\;{\overrightarrow{y}}_{k}^{{{{\rm{test}}}}}\right)}\end{array}$$

in Fig. 3i. This quantity allows the estimation of the dimensionality of the behavioral prediction.

#### Linear neural prediction using PCs of videos or keypoints

The mouse videos were reduced in dimensionality using SVD in blocks as described in ref. ^14^. The movie PCs were computed from the raw movie frames, and the top 500 PCs were used. Because the neural activity was recorded at a lower frame rate, the behavioral PCs were smoothed with a Gaussian filter of width 100 ms and then resampled at the neural timescale. We subtracted each behavioral PC by its mean and divided all PCs by the s.d. of the top behavioral PC.

A linear model called reduced rank regression (RRR) was used to predict neural PCs (*Y*) from the behavioral PCs or the corrected keypoint traces (*X*). RRR is a form of regularized linear regression, with the prediction weights matrix restricted to a specific rank^80^, reducing the number of parameters and making it more robust to overfitting. The RRR model is defined as

$$Y=XB{A}^{\top }$$

Like in ridge regression, the identity matrix times a *λ* constant can be added to the input covariance *X* for regularization. We set *λ* = 1 × 10^−^^6^. Training data were then used to fit the *A* and *B* coefficients in closed form; a rank of 128 was used for predicting from the movie PCs and a rank of 21 was used for predicting from the keypoints.

#### Neural prediction using a deep network

A multilayer network model was fit to predict neural activity from the movie PCs or the corrected keypoint traces using pytorch^34^ (Fig. 3a). The deep network model consisted of a core module and a readout module. The core module consisted of a fully-connected layer with the same dimensionality as the number of keypoints, a one-dimensional convolutional layer with ten filters (temporal convolution), a ReLU nonlinearity, two fully-connected layers with ReLU nonlinearities, the first with dimensionality of 50 and the second with dimensionality of 256. The 256-dimensional output of the core module is termed the ‘deep behavioral features’ of the model. The readout module of the network was one fully-connected layer, with a dimensionality of size 128 when predicting the neural PCs, or size equal to the number of neurons when predicting single neuron activity (when the number of neurons predicted was less than 200). The deep behavioral features, before entering the readout module, were subsampled at the timepoints coincident with the neural activity frames, because the videos were recorded at 50 Hz, while the neural activity was recorded at 3 Hz.

The deep network model was fit on the training data using the optimizer AdamW with LR of 1 × 10^−1^, weight decay of 1 × 10^−^^4^ and 300 epochs^81^, and the LR was annealed by a factor of 10 at both epochs 200 and 250. When fewer than 2,000 neurons were fit, the LR and weight decay were reduced by a factor of 10 to reduce overfitting. When fewer than 1 h of training timepoints were used, the LR and weight decay were reduced by a factor of 2, and the number of epochs was reduced by 100 to reduce overfitting. Each training batch consisted of a single training segment, with an average of length 10 min, and there were ten batches per recording. The model was then applied to the test segments to compute variance explained.

We varied various parameters of the network to approximately determine the best network architecture for neural prediction (Extended Data Fig. 4). We varied the number of units in the last layer of the core module, the ‘deep behavioral features’, from 1 to 1,024 (Extended Data Fig. 4a), and the number of convolution filters (Extended Data Fig. 4e). We varied the number of fully-connected layers with ReLU nonlinearities in the core module, each with dimensionality of 50 other than the last layer which was fixed at 256 dimensions (Extended Data Fig. 4b). We also varied the number of fully-connected layers in the readout module, with each layer having 128 dimensions and a ReLU nonlinearity, other than the last layer which had no output nonlinearity (Extended Data Fig. 4c). Next, from the original architecture described above, we removed components, such as the first fully-connected layer and some of the ReLU nonlinearities (Extended Data Fig. 4d).

#### Scaling of performance with neurons and timepoints

In Fig. 3g–i, we quantified the prediction performance as a function of the number of neurons and timepoints. For this analysis, we predicted using either a fraction of the neurons or a fraction of the training timepoints, while always keeping the test timepoints fixed. The variance explained was computed for each neuron, averaged across all neurons in the subset and then normalized by the explainable variance averaged over the neurons in the subset.

### Neural activity clustering and sorting

We identified groups of coactive neurons using scaled *k*-means clustering^70^. Compared to regular *k*-means, scaled *k*-means fits an additional variable *λ*_*i*_ for each neuron *i* such that

$$\begin{array}{r}{\overrightarrow{x}}_{i}={\lambda }_{i}{\mu }_{{\sigma }_{i}}+{{{\rm{noise}}}}\end{array}$$

where ${\overrightarrow{x}}_{i}$ is the activity vector of neuron *i*, *σ*_*i*_ is the cluster assigned to neuron *i* and *μ*_*j*_ is the activity of cluster *j*. Like regular *k*-means, this model is optimized by iteratively assigning each neuron to the cluster that best explains its activity, and then re-estimating cluster means. We ran scaled *k*-means clustering with 100 clusters on *z*-scored neural activity. Example clusters are shown in Fig. 4c and Extended Data Fig. 6. The activity of the neurons in each cluster was averaged to obtain a cluster activity trace (Fig. 4b). To obtain the cluster prediction from the deep behavioral model, we averaged the prediction of each neuron in the cluster (shown in gray in Fig. 4b), and then correlated this prediction with the cluster activity trace to obtain an *r* value for each cluster.

To quantify how spread out each cluster is in the recording field of view, we computed a locality index for each cluster. We defined the locality index as the Kullback–Leibler (KL) divergence between the cluster’s discretized spatial distribution in the recording field of view and the discretized spatial distribution of all neurons, using a discretization of 200 μm. We then correlated the locality index with the correlation of each cluster with its prediction (Fig. 4d,e).

### Fitting a discrete HMM

We fit a hidden Markov model (HMM) to the deep behavioral features ${\{{z}_{t}\}}_{t}$, where *t* is a time-step for temporal features that were downsampled ten times from 50 Hz to 5 Hz^43^. We also fit the same models to the keypoint data. All fitting procedures were the same, except for the choice of the variance term, which depends on the number of features (30 for the 11 keypoints from the Facemap tracker and 256 for the deep behavioral features) in the way described below. The HMM state dynamics are given by

$$\begin{array}{rc}{{{\rm{Prob}}}}({h}_{0}=i)&={b}_{i}\\ {{{\rm{Prob}}}}({h}_{t+1}=i| {h}_{t}=j)&={A}_{ji}\\ \mathop{\sum}\limits_{i}{b}_{i}&=1\\ \mathop{\sum}\limits_{i}{A}_{ji}&=1\end{array}$$

where *b*_*i*_ represents the probability of starting the Markov chain in state *i*, while *A*_*ji*_ represents the probability of transition from state *j* to state *i*. In all experiments, we chose the number of states to be 50, and we saw similar results with fewer (10) or more (200) states. Because our goal is to understand the pattern of dynamics of the deep behavioral features, we did not attempt to infer the ‘optimal’ number of states and do not believe the data lends itself easily to such an estimation.

In addition to state dynamics, an HMM has an ‘observation’ or ‘emission’ model, which declares the probability of observing some data sample *z*_*t*_ for each possible state *h*_*t*_:

$$\begin{array}{rc}{{{\rm{Prob}}}}({z}_{t}| {h}_{t}=i)&={{{\mathcal{N}}}}({z}_{t}| {C}_{i},\sigma )\end{array}$$

where *C*_*i*_ and *σ* are the mean and s.d. of the Gaussian observation model, respectively. This completes the model specification. We optimized this model in Pytorch using an improved, nonstandard optimization scheme, which routinely optimized the model better compared to alternative optimization methods such as expectation maximization.

Our optimization scheme consists of (1) optimizing the model log-likelihood directly as a function of its parameters using the automated differentiation from pytorch and (2) using initializations and reparametrizations of the HMM parameters that improve stability.

The log-likelihood of the HMM can be computed based on the forward pass of the ‘forward-backward’ algorithm. Following the convention of ref. ^44^, we define *α*(*h*_*t*_) = Prob(*z*_1_, *z*_2_, …, *z*_*t*_, *h*_*t*_). We can then define recursion equations for computing

2 $$\alpha ({h}_{t})={{{\rm{Prob}}}}({z}_{t}| {h}_{t})\mathop{\sum}\limits_{{z}_{t-1}}\alpha ({z}_{t-1}){{{\rm{Prob}}}}({z}_{t}| {z}_{t-1})$$

The full log-likelihood of the data can then be computed based on *α*(*h*_*T*_), where *T* is the last timepoint, by observing that

$$\begin{array}{ll}\mathop{\sum}\limits_{i}\alpha ({h}_{T}=i)&=\mathop{\sum}\limits_{i}{{{\rm{Prob}}}}({z}_{1},\ldots ,{z}_{T},{h}_{T}=i)\\ &={{{\rm{Prob}}}}({z}_{1},\ldots ,{z}_{T})\end{array}$$

Because the dependence of *α*_*t*+1_ on *α*_*t*_ can be written in closed form, we can see that it is differentiable. After taking the logarithm and replacing the probabilities with the model equations, Eq. (2) becomes

3 $${\alpha }_{i}(t)=-\parallel {z}_{t}-{C}_{i}{\parallel }^{2}/{\sigma }^{2}-0.5n\sigma +C+ {\log}\left(\mathop{\sum}\limits_{j}\exp ({\alpha }_{j}(t-1)){A}_{ji}\right)$$

where ${\alpha }_{i}(t)=\log (\alpha ({h}_{t}=i))$, *C* is a constant and *n* is the number of dimensions of the data. This formulation allows us to use the automatic differentiation from pytorch to optimize the HMM model directly, without inferring states first like in the expectation maximization method. Additionally, we note that we used the ‘logsumexp’ function from pytorch to compute the second half of Eq. (3), which has the advantage of being stable to exponentiation.

We re-parametrized the transition matrix *A* with a ‘log-transition’ matrix *Q* by

$${A}_{ji}=\exp ({Q}_{ji})/\mathop{\sum}\limits_{{i}^{{\prime} }}\exp ({Q}_{j{i}^{{\prime} }}).$$

This has the advantage of removing the constraint of positivity of *A*_*ji*_ and the constraint of summing to 1 of the rows of *A*. We initialized the log-transition matrix with *Q*_*ii*_ = 3 and *Q*_*ij*_ = 0 when *i* ≠ *j*, and we initialized the parameters *C*_*i*_ of the observation model with random samples from the data. For setting *σ*, we made the choice of freezing it to a fixed value for each dataset. This was because of the dependence of the log-likelihood on the number of observation dimensions *n* in Eq. (3). Because *n* is quite different between the keypoints and the deep behavioral features, the relative contribution of the observation term to the likelihood would be different if we set or learned *σ* to be the same in the two cases, potentially biasing the model to rely more or less on the internal hidden states *h*_*t*_. Instead, we fix *σ*^2^ to be proportional to the summed variance of *z*_*t*_, and we set it to 1 for the deep behavioral features, and 30/256 for the keypoints model. This ensures an approximately equal weighting of the observation term into the likelihood model. We note that the properties of the fitted HMM were not substantially different when *σ*^2^ was set to the same value for the keypoints and deep behavioral features, but the quality of the samples simulated from the HMM degraded if *σ*^2^ was too low.

### Properties of the discrete HMM

The inferred states were determined with the Viterbi algorithm, which finds the most likely hidden states. We simulated states by drawing initial states from the categorical distribution with parameters *b*_*i*_, and then running the forward dynamics and drawing states from the conditional distributions Prob(*h*_*t*+1_ = *i*∣*h*_*t*_ = *j*) = *A*_*ji*_.

State lifetimes were defined as $-\log (1-{A}_{ii})$, and they correspond to the mean durations of staying in state *i*. To compute transition sparsity and other metrics, we set self-transitions *A*_*ii*_ = 0 and renormalized the rows. Formally, we defined a transition matrix ${B}_{ji}={A}_{ji}/{\sum }_{{i}^{{\prime} }\ne j}{A}_{j{i}^{{\prime} }}$ when *j* ≠ *i* and *B*_*ii*_ = 0. This is the matrix shown in Fig. 5d,e used for the analyses in Fig. 5i–n. The states were sorted using the Rastermap algorithm on the matrix *B*^46^. Specifically, this involves maximizing the similarity of the reordered transition matrix to the matrix given by *F*_*ji*_ = −log((*i* − *j*)^2^) when *j* < *i* and 0 otherwise. Thus, the model attempts to put the highest probabilities close to the diagonal, and specifically above the diagonal, because they do not count if they are below the diagonal. For more details, see the rastermap repository at github.com/MouseLand/rastermap.

The transition sparsity was computed by sorting the rows of the matrix *B* in descending order, and computing a cumulative sum over each row. ‘Near’ states were defined as the five states *i* with the highest probability *A*_*ji*_ for a given *j*. Reverse transitions were computed for each state based on its near states. Similarly, we computed the two-state forward and backward transitions. Forward sequences were computed based on the most likely inferred states, by counting the number of increasing sequences of each length. Note this depends on the initial Rastermap sorting of states to define a meaningful order.

### Statistics and reproducibility

No statistical method was used to predetermine sample size, but our sample sizes are similar to those reported in previous publications^14,16,17^. We performed Wilcoxon signed-rank tests, which do not require the data to be normal. No data were excluded from the analyses. There were no experimental groups so there was no randomization necessary. Data collection and analysis were not performed blind to the conditions of the experiments.

### Reporting summary

Further information on research design is available in the Nature Portfolio Reporting Summary linked to this article.

## Online content

Any methods, additional references, Nature Portfolio reporting summaries, source data, extended data, supplementary information, acknowledgements, peer review information; details of author contributions and competing interests; and statements of data and code availability are available at 10.1038/s41593-023-01490-6.

### Supplementary information

Supplementary Information Supplementary Tables 1 and 2.

Reporting Summary

Supplementary Video 1 Mouse face video showing keypoint tracking of the test mouse, 0.33× real time.

Supplementary Video 2 Mouse face videos and keypoint tracking from other lab data (Fig. 2) using the base model, and the scratch and fine-tuned models trained with ten frames, 0.4–0.7× real time.

Supplementary Video 3 Mouse face video and keypoint tracking from a freely-moving mouse (Extended Data Fig. 3) using the base model, and the fine-tuned model trained with ten frames, 17× real time.

Supplementary Video 4 Visualization of four example states from the HMM shown in Fig. 5, with 15 instances of each state shown. Up to 5 s of time is shown in total at a speed of 0.5× real time, with 0.5 s before the state starts shown (fade in from gray), and 1.0 s after the state ends is shown, if the state is less than 3.5 s (fade out from gray).

## Data Availability

The data generated in this study is available on figshare: 10.25378/janelia.23712957.

## Code availability

Facemap was used to perform all analyses in the paper, the code and GUI are available at https://www.github.com/mouseland/facemap, including video and code-based guides for using the software. Scripts for running the analyses in the paper are available at https://github.com/MouseLand/facemap/tree/main/paper.
