# Supplementary material for: Facemap: a framework for modeling neural activity based on orofacial tracking
Source: Nat Neurosci. 2023 Nov 20;27(1):187–95. doi: 10.1038/s41593-023-01490-6 (PMC10774130; doi:10.1038/s41593-023-01490-6)
Supplement: Supplementary file 1 — Supplementary Tables 1 and 2. [file 41593_2023_1490_MOESM1_ESM.pdf]

nature neuroscience

Technical Report

https://doi.org/10.1038/s41593-023-01490-6

Facemap: a framework for modeling neural activity based on orofacial tracking

In the format provided by the authors and unedited

Supplementary Information

	Facemap	DeepLabCut (ResNet50)	DeepLabCut (Mobilenet)	SLEAP (default)	SLEAP (c=32)
# Trainable parameters	2,148,271	23,904,160	396,128	1,953,567	31,406,127
# downsample blocks	5	5	4	5	5
# upsample blocks	2	1	1	4	4

Table 1: **Network architecture details of the Facemap tracker, DeepLabCut and SLEAP models.** Number of trainable parameters and the number of downsample and upsample blocks used for each model. Facemap's network is smaller than the standard "U-Net" architecture which is used in SLEAP [31,32]: it uses fewer channels per convolutional layer and has two upsampling blocks rather than four, instead predicting location refinement maps to obtain predictions at the full resolution (Figure 1a).

	Facemap	DeepLabCut (ResNet50)	DeepLabCut (Mobilenet)	SLEAP (default)	SLEAP (c=32)
V100	**327.2 ± 0.6**	150.1 ± 0.6	211.2 ± 1.7	80.0 ± 0.4	72.4 ± 0.4
A100	**343.1 ± 5.5**	189.1 ± 1.1	243.8 ± 9.2	110.6 ± 1.4	103.0 ± 1.0
RTX 2080 Ti	**315.9 ± 1.5**	132.5 ± 0.7	200.0 ± 0.5	88.9 ± 0.2	77.7 ± 0.2
Tesla T4	**272.9 ± 0.2**	98.9 ± 1.0	203.4 ± 2.6	75.4 ± 0.6	64.1 ± 0.2
Quadro RTX 8000	**332.1 ± 0.2**	138.9 ± 1.6	202.4 ± 1.3	92.7 ± 0.4	78.6 ± 0.5
Colab	**250.0 ± 4.5**	98.5 ± 0.2	163.5 ± 0.5	110.5 ± 1.3	92.5 ± 0.8
CPU	33.5 ± 1.2	19.2 ± 0.1	**43.2 ± 1.2**	37.8 ± 1.0	19.6 ± 0.2

Table 2: **Processing speed of pose estimation models on different GPUs.** A sample image of size 256 $\times$ 256 pixels was used as input for Facemap, DeepLabCut (ResNet50), DeepLabCut (Mobilenet), SLEAP (default) and SLEAP (c=32) models. The time taken for a forward pass through the network and post-processing steps was used to obtain processing speed for batch size of 1. A total of 1024 repetitions and 10 runs were used to obtain the average processing speed. The number of CPU cores/slots used for different GPUs were as follows: A100 (48 slots, 40GB/slot), V100 (48 slots, 30GB/slot), RTX 2080 Ti (40 slots, 18GB/slot), Tesla T4 (48, 15GB/slot) and Quadro RTX 8000 (40 slots, 18GB/slot). The CPU used was 3.0GHz Intel Cascade Lake(Gold 6248R) with 768GB and 8 slots used for timing on CPU only. Models were also run on Google Colab's GPU.
