# Supplementary material for: Facemap: a framework for modeling neural activity based on orofacial tracking
Source: Nat Neurosci. 2023 Nov 20;27(1):187–95. doi: 10.1038/s41593-023-01490-6 (PMC10774130; doi:10.1038/s41593-023-01490-6)
Supplement: Supplementary file 2 — Reporting Summary [file 41593_2023_1490_MOESM2_ESM.pdf]

nature portfolio

Corresponding author(s): Carsen Stringer

Last updated by author(s): 2023/09/11

Reporting Summary

Nature Portfolio wishes to improve the reproducibility of the work that we publish. This form provides structure for consistency and transparency in reporting. For further information on Nature Portfolio policies, see our Editorial Policies and the Editorial Policy Checklist.

Statistics

For all statistical analyses, confirm that the following items are present in the figure legend, table legend, main text, or Methods section.

n/a	Confirmed	
☐	☒	The exact sample size (n) for each experimental group/condition, given as a discrete number and unit of measurement
☒	☐	A statement on whether measurements were taken from distinct samples or whether the same sample was measured repeatedly
☐	☒	The statistical test(s) used AND whether they are one- or two-sided<br>*Only common tests should be described solely by name; describe more complex techniques in the Methods section.*
☒	☐	A description of all covariates tested
☒	☐	A description of any assumptions or corrections, such as tests of normality and adjustment for multiple comparisons
☐	☒	A full description of the statistical parameters including central tendency (e.g. means) or other basic estimates (e.g. regression coefficient) AND variation (e.g. standard deviation) or associated estimates of uncertainty (e.g. confidence intervals)
☒	☐	For null hypothesis testing, the test statistic (e.g. F, t, r) with confidence intervals, effect sizes, degrees of freedom and P value noted<br>*Give P values as exact values whenever suitable.*
☒	☐	For Bayesian analysis, information on the choice of priors and Markov chain Monte Carlo settings
☒	☐	For hierarchical and complex designs, identification of the appropriate level for tests and full reporting of outcomes
☐	☒	Estimates of effect sizes (e.g. Cohen's d, Pearson's r), indicating how they were calculated

Our web collection on statistics for biologists contains articles on many of the points above.

Software and code

Policy information about availability of computer code

Data collection	Scanimage software v2022.1.0 (open source) was used to collect calcium imaging data from awake mice using a two-photon mesoscope (Thorlabs 2PRAM microscope). BIAS software (open source, github version: https://github.com/janelia-idf/bias) and custom code were used to collect mouse face videos.
Data analysis	We processed all of the raw calcium imaging data using our suite2p package https://github.com/mouseland/suite2p (version 0.9.4). We processed the mouse face videos using our Facemap software package, available at https://github.com/mouseland/facemap. The code for running several of the analyses in the paper is available at https://github.com/MouseLand/facemap/tree/dev/paper/code.<br>We ran the code with python=3.8.13, pytorch=1.11.0, numpy=1.23.3, scipy=1.9.1, pyqt5=5.15.7, pyqtgraph=0.12.0, opencv-pythonheadless=4.6.0.66, numba=0.56.2, tqdm=4.64.1 , pandas=1.5.0, and matplotlib=3.6.0.

For manuscripts utilizing custom algorithms or software that are central to the research but not yet described in published literature, software must be made available to editors and reviewers. We strongly encourage code deposition in a community repository (e.g. GitHub). See the Nature Portfolio guidelines for submitting code & software for further information.

Data

Policy information about availability of data

All manuscripts must include a data availability statement. This statement should provide the following information, where applicable:

- Accession codes, unique identifiers, or web links for publicly available datasets
- A description of any restrictions on data availability
- For clinical datasets or third party data, please ensure that the statement adheres to our policy

All data generated by the current study is available on figshare, DOI: 10.25378/janelia.23712957

Research involving human participants, their data, or biological material

Policy information about studies with human participants or human data. See also policy information about sex, gender (identity/presentation), and sexual orientation and race, ethnicity and racism.

Reporting on sex and gender	N/A
Reporting on race, ethnicity, or other socially relevant groupings	N/A
Population characteristics	N/A
Recruitment	N/A
Ethics oversight	N/A

Note that full information on the approval of the study protocol must also be provided in the manuscript.

Field-specific reporting

Please select the one below that is the best fit for your research. If you are not sure, read the appropriate sections before making your selection.

☒ Life sciences ☐ Behavioural & social sciences ☐ Ecological, evolutionary & environmental sciences

For a reference copy of the document with all sections, see nature.com/documents/nr-reporting-summary-flat.pdf

Life sciences study design

All studies must disclose on these points even when the disclosure is negative.

Sample size	We did not perform a sample-size calculation. We performed 16 neural recordings in 12 mice: 6 in sensorimotor cortical areas and 10 in visual cortical areas. This is sufficiently many mice, comparable to other studies of spontaneous neural activity (see citations 14, 16, 17).
Data exclusions	We did not exclude any data from the analyses.
Replication	We have used standard mouse-lines available from JAX and processed the data with an automated algorithm to avoid any personal biases. We used a standard commercial microscope (Thorlabs 2P-RAM microscope). We have also made all of the code available for analysis by others, and will make the data available to the public upon publication.
Randomization	Not relevant, there are no experimental groups.
Blinding	Not relevant, there are no experimental groups.

Reporting for specific materials, systems and methods

We require information from authors about some types of materials, experimental systems and methods used in many studies. Here, indicate whether each material, system or method listed is relevant to your study. If you are not sure if a list item applies to your research, read the appropriate section before selecting a response.

Materials & experimental systems

n/a	Involved in the study
☒	☐ Antibodies
☒	☐ Eukaryotic cell lines
☒	☐ Palaeontology and archaeology
☐	☒ Animals and other organisms
☒	☐ Clinical data
☒	☐ Dual use research of concern
☒	☐ Plants

Methods

n/a	Involved in the study
☒	☐ ChIP-seq
☒	☐ Flow cytometry
☒	☐ MRI-based neuroimaging

Animals and other research organisms

Policy information about studies involving animals; ARRIVE guidelines recommended for reporting animal research, and Sex and Gender in Research

Laboratory animals	TetO-GCaMP6s x Emx1-IRESCre mice (available as RRID:IMSR JAX:024742 and RRID:IMSR JAX:005628). These mice were male and female, and ranged from 2 to 12 months of age.
Wild animals	The study did not involve wild animals.
Reporting on sex	All data was aggregated across sex. We did not perform sex-based analyses because our questions were not related to sex-based differences in behavior or neural activity.
Field-collected samples	The study did not involve field samples.
Ethics oversight	All experimental procedures were conducted according to IACUC, ethics approval received from the IACUC board at HHMI Janelia Research Campus.

Note that full information on the approval of the study protocol must also be provided in the manuscript.
